# Data on gender representation in food and beverage print advertisements found in corner stores from Guatemala and Peru

**DOI:** 10.1186/s13104-021-05469-z

**Published:** 2021-02-16

**Authors:** Lucila Rozas, Peter Busse, Joaquin Barnoya, Alejandra Garrón

**Affiliations:** 1 Grupo de Investigación en Comunicación y Salud, Instituto de Investigación Científica, Universidad de Lima, 4600 Javier Prado Este Avenue, Tower A, 11th Floor, 15023 Lima, Peru; 2 Departamento de Investigación, Unidad de Cirugía Cardiovascular de Guatemala, 8-00 9th Avenue, 11th Zone, 01011 Guatemala City, Guatemala; 3 Fundación Interamericana del Corazón-Bolivia, Mariscal Santa Cruz Avenue, La Primera Building, Tower B, 10th Floor, Office No. 7, La Paz, Bolivia

**Keywords:** Food marketing, Gender, Children, Advertisement, Print media, Prevalence

## Abstract

**Objectives:**

Data on gender representation in food and beverage advertisements may allow for a better understanding of how the food industry is targeting different audiences based on gender. Nonetheless, scant research on food and beverage print advertising with a gender approach has been conducted. Therefore, we sought to assess the prevalence of gender focus in print advertisements found inside corner stores in two cities: Guatemala City, Guatemala, and Lima, Peru.

**Data description:**

We developed two complementary datasets as part of the study: (1) a dataset of digital photographs of 200 food and beverage print advertisements found in corner stores located near schools (100 ads per country selected according to criteria such as product type, image quality, and uniqueness); (2) a quantitative dataset with data of the content analysis of these photographs. We employed 19 variables to record the general information and gender assessment of the ads. These datasets should allow scholars and public officials to identify gender-specific marketing strategies of the food industry that might impact children’s and adolescents’ nutrition differently.

## Objective

The main objective was to assess the prevalence of gender focus in print advertisements found inside corner stores in two cities: Guatemala City, Guatemala, and Lima, Peru. Content analysis data assessing gender representation in point of sale (POS) food and beverage print advertisements are relevant to understand how the food industry might be targeting different audiences based on gender. Previous research shows that gender is associated with food consumption [1–5] and that the food industry has been using this knowledge to create gender-specific advertising strategies [6–15].

However, there is a dearth of studies regarding the prevalence of gender focus in print food advertisements, especially in low-middle-income countries such as Guatemala and Peru. This is surprising considering that exposure to food and beverage advertising is one of the risk factors linked to increased BMI (body mass index) in children [16] and that obesity rates are on the rise in this region, particularly in younger populations [17].

The resulting data are unique since they include two complementary datasets for assessing gender focus in food and beverage print advertisements. Researchers and public officials can use these datasets to evaluate how the food industry uses gender-specific elements in print advertising and how they may be reproducing gender stereotypes that could cause unequal health outcomes, mostly among children and adolescents.

## Data description

The data from this project and its supporting documentation are stored in the Inter University Consortium for Political and Social Research (ICPSR) open data repository (openICPSR) and includes two complementary datasets [18]. First, a dataset with 200 photographs of food and beverage print ads found inside corner stores around four schools in Guatemala City and Lima (100 ads per country). Saturation defined the sample size as, at some point during data collection, researchers did not find new print ads to photograph. Second, a quantitative dataset with content analysis data of the 200 photographs of print ads [18].

We developed the image dataset during the fieldwork stage, which took place between October 2018 and December 2018. This stage entailed various procedures for collecting data. First, four neighborhoods in each city were selected according to pre-established criteria (presence of at least one school with an average population of 400 students; safety and accessibility of the area; presence of corner stores; presence of print advertisement; socioeconomic status). Then, researchers walked a radius of 400 m around each school in every neighborhood, since this is the distance students would typically walk to get food [19, 20]. The research team marked on digital maps the geolocation of the corner stores where to take photographs of the ads using smartphones and a Global Positioning System (GPS) device (Garmin brand). Finally, each researcher visited one of the areas for capturing the photographs and then selected each photograph according to image quality and uniqueness to include it in the sample. The image records are stored in a compressed image format (.JPG) and each one has a numerical code that matches a unique record in the quantitative dataset.

Before conducting fieldwork, the team defined a list of food and beverage categories for researchers to know what ads they should photograph, based on previous studies [19, 21]. This list and other supporting documentation that describes the procedures of obtention of the image dataset is in the repository [18], in plain text format (.TXT) (see data file 1 and data file 2 described in Table 1).

**Table 1:** Overview of data files/data sets

Label	Name of data file/data set	File types (file extension)	Size	Data repository and identifier (DOI or accession number)	Date of last modification (DD/MM/YYYY)
Data set 1	Image dataset of food and beverage print ads in Guatemala City and Lima	Joint Photographic Expert Group files (.jpeg)	956 MB	Open ICPSR (https://doi.org/10.3886/E122441V5)	15/10/2020
Data set 2	Quantitative datasets in English and Spanish with coded data of the assessment of gender representation in print advertisement	Comma Separated Values files (.csv)	19.7 KB	Open ICPSR (https://doi.org/10.3886/E122441V5)	23/09/2020
Data file 1	Data obtention process flowchart	Portable Document Format (.pdf)	110.2 KB	Open ICPSR (https://doi.org/10.3886/E122441V5)	29/10/2020
Data file 2	Guide photographs GenderAds	Plain Text file (.txt)	3 KB	Open ICPSR (https://doi.org/10.3886/E122441V5)	22/09/2020
Data file 3	Fieldwork guide GenderAds	Plain Text file (.txt)	6.7 KB	Open ICPSR (https://doi.org/10.3886/E122441V5)	22/09/2020
Data file 4	List of image authorship Guatemala	Comma Separated Values file (.csv)	4.5 KB	Open ICPSR (https://doi.org/10.3886/E122441V5)	22/09/2020
Data file 5	List of image Authorship Peru	Comma Separated Values file (.csv)	4.3 KB	Open ICPSR (https://doi.org/10.3886/E122441V5)	22/09/2020
Data file 6	POS checklist instrument GenderAds	Plain Text file (.txt)	2.6 KB	Open ICPSR (https://doi.org/10.3886/E122441V5)	17/09/2020
Data file 7	Upload and Analysis Guide GenderAds	Plain Text file (.txt)	5.3 KB	Open ICPSR (https://doi.org/10.3886/E122441V5)	22/09/2020
Data file 8	Variables definitions print ads	Plain Text file (.txt)	5.6 KB	Open ICPSR (https://doi.org/10.3886/E122441V5)	22/09/2020

The quantitative dataset with content analysis data of the 200 photographs of print ads was developed by an expert marketing firm with previous experience working with the team in Guatemala. This dataset is stored in a comma-separated values (.CSV) format and comprises the aggregated data of Guatemala and Peru. The team developed a coding tool with 19 variables, as well as a codebook in plain text format [18], to record general and gender assessment information of each ad in the image dataset. The marketing firm and the research team had periodic discussions during analysis to validate the process and ensure consistent results. The process of data obtention of the image and quantitative datasets is explained in a flowchart that is stored in the repository in a portable document format (.PDF) [18].

## Limitations

The sample used in this study is limited to advertisements found in bodegas located in Guatemala City and Lima, and therefore the analysis of these data would only allow obtaining a partial picture of the gender representation in food and beverage print advertisements. Moreover, even when the content analysis initially included a qualitative approach, the datasets only report the final assessment of the ads. Furthermore, the process of geolocating and developing digital maps of the corner stores for conducting fieldwork was different in Guatemala and Peru. Due to limited resources and equipment availability, the team in Peru used smartphones and Google Maps, while the team in Guatemala used a Global Positioning System device and the free Geographic Information System software QGIS v2.18. Nonetheless, the difference in both procedures did not affect the quality of data collection in both countries, as the maps were developed for location reference and not as an output for analysis. Finally, considering that food marketing techniques change fast due to regulations and other factors, the data will be available openly available for reuse for a maximum period of 5 years.

## Data Availability

The data described in this Data note can be freely and openly accessed on the openICPSR repository under reference number openicpsr-122441, https://doi.org/10.3886/E122441V5. Please see Table 1 and reference number [12] for details and links to the data.

## Abbreviations

POS: Point of sale
BMI: Body mass index
ICPSR: Inter University Consortium for Political and Social Research
GPS: Global positioning systemec1
